# A state of limbo – in transition between two contexts: Health assessments upon arrival in Sweden as perceived by former Eritrean asylum seekers

**DOI:** 10.1177/1403494815576786

**Published:** 2015-07

**Authors:** Robert Jonzon, Pille Lindkvist, Eva Johansson

**Affiliations:** 1 The Public Health Agency of Sweden, Sweden; 2 Department of Public Health and Clinical Medicine, Umeå University, Sweden; 3 Center for Family Medicine (CeFAM), Karolinska Institute, Sweden; 4 Department of Public Health Sciences (IHCAR), Karolinska Institute, Sweden; 5 Nordic School of Public Health, Sweden

**Keywords:** Asylum seekers, migration, migrant health, health assessments, grounded theory

## Abstract

*Background:* National statistics have shown that only about 40% of asylum seekers actually attend the optional health assessment offered upon their arrival in Sweden, but the reasons for this have not been fully explored. Health assessments for newly arrived asylum seekers have become a regular practice in most EU countries, but what is performed, how it is organized and whether it is mandatory or not varies between countries. *Aim*: The aim of the study was to explore and improve our understanding of how former asylum seekers from Eritrea perceived and experienced the health assessment during their asylum-seeking process. *Methods*: We used a qualitative research approach guided by grounded theory. Semi-structured interviews were conducted with 11 former asylum seekers from Eritrea. Data were analysed based on constant comparative analysis. *Findings*: The asylum seekers expressed feelings of ambiguity and mistrust and felt that they were seen only as objects by the Swedish healthcare system during their asylum-seeking process. Poor communication and inability to overcome language and cultural barriers seemed to be the most important findings in the narratives. The core category was defined as ‘A state of limbo – in transition between two contexts’. ***Conclusions*: There are reasons to believe that these issues with communication negatively affected both the quality of the health assessment and the number of asylum seekers attending the health assessment. Improved communication by the authorities towards the asylum seekers is, therefore, of vital importance.**

## Background

Health assessments for newly arrived asylum seekers have become a regular practice in most EU countries [1]. National statistics in Sweden, however, show that only approximately 40% of the asylum seekers actually attend a health assessment [2]. The reasons for the low attendance have not been fully explored.

In a recent Swedish study, the researchers attempted and failed to identify individual asylum seekers who had actively rejected the invitation to a health assessment [personal communication, F. Kalengayi Nkulu]. Limited research has been carried out on how asylum seekers in the EU and elsewhere perceive primary healthcare services, including health assessments, and how their experiences might influence the willingness to utilize these services [3,4].

By law, health assessments are to be offered to asylum seekers upon their arrival in Sweden [5]. The Swedish Migration Board is obliged to inform them about this right, which should be followed by an invitation from the healthcare system including a clarification on the purpose of the health assessment, that it is optional, that an interpreter can assist if needed and contact details for the caregiver [6].

During the asylum-seeking process in Sweden, immigrants have the option to stay either in accommodation centres provided by the migration authority or to stay in private homes with relatives or friends. This might lead to a frequent change of residence. Also, during this time, which for some could last for years, there is no provision of language training.

Health-related concerns in relation to immigration appear to have mostly focused on the transmission of infectious agents [7]. To what extent asylum seekers and their families are affected by infectious diseases is not fully known [8], but many asylum seekers and refugees come from countries where severe infections are endemic and diseases such as hepatitis, tuberculosis (TB) and HIV are far more common than in Sweden [9]. The number of new HIV cases reported in Sweden has been relatively stable over time, with a slowly increasing trend since 2003 mainly due to the immigration of people infected prior to their arrival in Sweden [10]. Several studies of immigrant populations have shown that migrants in general tend to come late for their first HIV test and this leads to subsequent delays in diagnosis of HIV and treatment [11,12]. In Sweden, the health assessment should be seen as part of the systematic attempt by which the authorities are trying to tackle the spread of infectious diseases [13]. However, some researchers have found that the predominant health concerns experienced by adult asylum seekers are various kinds of psychosomatic disorders – which seem to be connected to traumatic stress ahead of and during the asylum-seeking process [4,14] – while others suggest a linkage between migration and diabetes [15]. The Swedish regulations state that the purpose of the health assessment is not only to detect contagious diseases but any disease that needs immediate attention that cannot be deferred [6].

During the recent 2 years Eritrean nationals comprise the second largest group applying for asylum in Sweden [16]. As they constitute a large newly immigrated population in Sweden it was found relevant to improve understanding on their experience of the health assessment.

The aim of this study was to explore and improve understanding of how asylum seekers from Eritrea perceived and experienced the health assessment during their asylum-seeking process in Sweden.

## Methodology

### Design

We used a qualitative research approach guided by grounded theory as originally developed by Glaser and Strauss [17]. Grounded theory offers a systematic set of procedures that can be used to create theoretical concepts explaining social phenomena and processes and to inductively build a hypothesis, model or theory from data grounded in reality. An emergent design was used in which the data collection and analysis took place in parallel. The researchers adjusted the inquiry as the study progressed based on what had been learned in the study up to that point. The intention was to build a model or theory that could explain the process of the phenomenon under study, in this case the influences on the asylum seekers’ perception of the health assessment. Grounded theory was found to be an appropriate approach throughout the study because it is based on symbolic interactionism that is suitable when interpreting social processes [18,19].

### Study setting and participants

The interviews took place in a language school for immigrants. The school was situated in the neighbourhood of the homes of most participants and the interviews were held in the evenings when no ordinary school activities took place. This was considered to be a neutral place where the participants could feel comfortable and safe.

The participants were purposively selected with the assistance of a trusted person who was responsible for the local introduction programme for refugees and other immigrants. The inclusion criteria were that the participants should be of Eritrean origin and have lived in Sweden for less than 5 years. Eight persons were initially recruited, four women and four men. Three more persons, one woman and two men, were later theoretically sampled to ensure data saturation. They all held a residence permit and resided in Stockholm at the time of the interviews. The age of the selected participants ranged from 27 to 43 years and they had lived in Sweden between 2.5 and 5 years.

### Research team

The research team represented expertise in public health and qualitative research methods with research experiences from Sweden and countries in Africa and Asia. Additionally, the team comprised competences in anthropology (R.J.), global health (E.J.), epidemiology and infectious diseases (P.L.).

### Data collection

The data were collected during the spring of 2013 using individual semi-structured interviews. Such interviews have been shown to be suitable when addressing sensitive questions on health issues [18–20]. A thematic interview guide, based on the literature and the authors’ personal and professional experience, was used and all interviews were digitally recorded and lasted for approximately 1 hour.

The first author conducted the interviews. He was assisted by one of the other researchers, who acted as an observer. The interviews were mainly held in Swedish but with support from a specially trained interpreter whose mother tongue is Tigrinya and who translated when necessary.

The interview questions were initially broad, but probing and follow-up questions led to more focused answers and a deeper understanding of the data. Each of the interviews ended with a member check where the researcher read back a summary of the interview. By doing so, the informants could adjust or alter their comments immediately while the topics discussed were still fresh in their mind. Their comments were recorded at the same time.

The researcher, the observer and the Tigrinya-speaking interpreter discussed the interview process and defined topics of special interest for further exploration after each interview. Memos were written to record information and the reflections on the process.

### Data analysis

**Figure 1.**
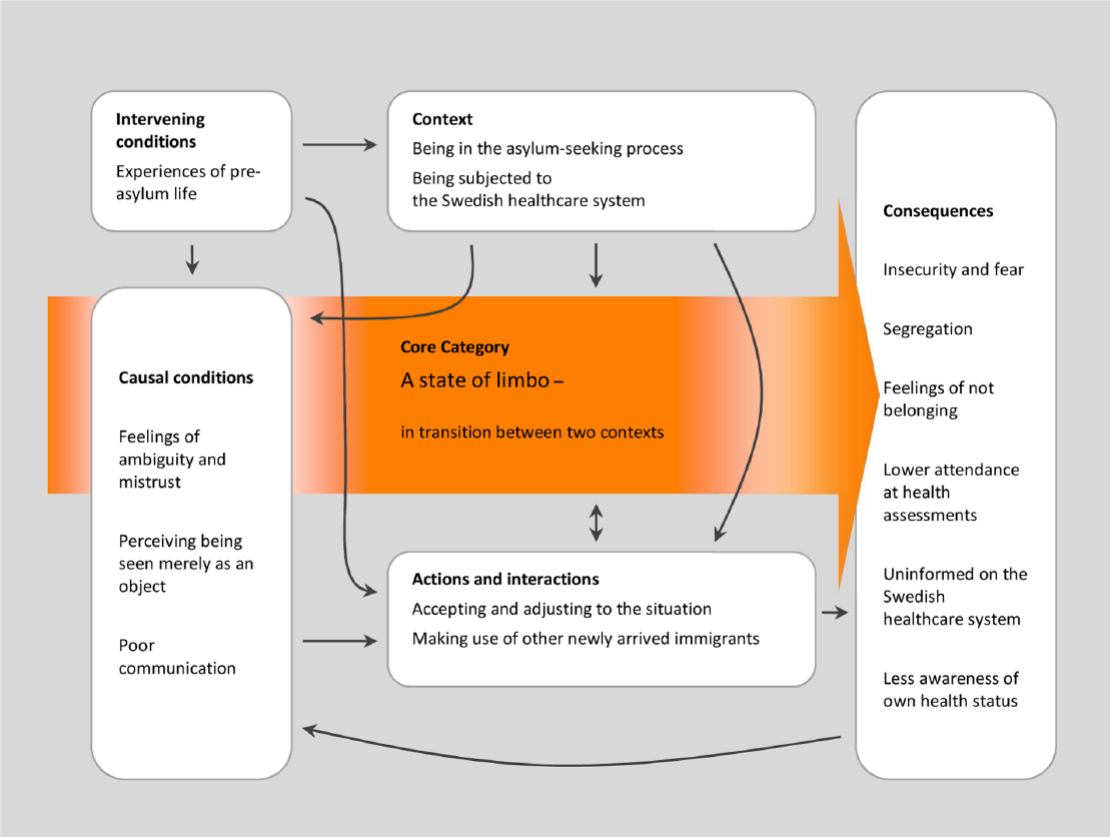
The organisation of concepts and categories using the paradigm model.

The interviews were transcribed verbatim in Swedish and analysed according to grounded theory principles, which means open, axial and selective coding (hand coding) using constant comparisons. The paradigm model was used as a framework for the integration of categories and concepts (see Figure 1). The model required the identification of factors (categories and other concepts) that represent context, causal conditions, intervening conditions, action and interaction strategies and how categories and underlying concepts relate to each other and to the core category [18,19].

The properties (characteristics of categories) and dimensions (variations within properties) of each category were identified. This contributed to a broader understanding of the data by enabling a wider exploration of the categories. An example of a category with developed properties and dimensions is presented in Table I.

**Table I.** Properties and dimensions of categories related to intervening conditions.

Category	Property	Dimension (variety)
Experiences of pre-asylum life	Environmental aspects	Urban_______________Rural
		Stationary__________Mobile
		Peace_____________Conflict
	Healthcare	Available_____ Not available
	Preventive measures	Sufficient_______Insufficient
	Education	High________________ Low
	Job/military service	Yes__________________No

The analysis was performed by the first author in collaboration with the co-authors. The first step of the analysis took place immediately after the interviews when the interviewer and the observer discussed and analysed the data. Crucial issues that were identified were then addressed in the subsequent interviews as part of the emergent design in order to develop and refine the quality of the data [21].

## Ethical considerations

The study was approved by the Regional Ethical Review Board in Stockholm (reg. no. 2012/1893/31/5).

Interviewees were given written and oral information about the background and purpose of the study. They were informed that participation was voluntary, that they could terminate their participation at any time without any negative consequences and that confidentiality was assured. The information given to the study participants also covered reasons why the interviews were to be recorded. Informed written and oral consent was obtained. To protect the integrity of the respondents, the citations are confined to the sex and age of the participant.

## Findings

One core category and several subordinate categories were identified. The paradigm model was used as a framework for the findings and these illustrate how the core category and the other categories interrelate (Figure 1). In this study, the core category was identified as a state of limbo – in transition between two contexts. Poor communication, a central theme throughout the narratives, contributed to feelings of fear, ambiguity and exclusion. Quotations that illustrate the actual views of the informants are included in order to strengthen the trustworthiness of the study.

Categories were sorted under headings used in the paradigm model.

### A state of limbo

The core category in this study was identified as a state of limbo – in transition between two contexts. Limbo, or liminality, encapsulates the feelings and experiences communicated by the informants, including separation, vulnerability, uncertainty, unpredictability, lack of trust, social exclusion and marginalization. Furthermore, it captures the inability to overcome the language and cultural barriers that seemed to cause the state of limbo. This failure to communicate began at the very beginning of the asylum-seeking process. The initial information about health-related issues, such as the right to undergo a health assessment, was to be given to the newly arrived asylum seekers by the migration authority, but only few of the informants in this study were able to recall this information being provided to them. Some said they had been given information about the health assessment, while others denied it. Whether this information was not given at all or was just drowned out by all of the other information that was said to have been given at the same time is not possible to say.

Almost all of the informants in this study had received a written invitation from the Swedish healthcare system. However, because the invitation was written in Swedish, many did not understand its contents or why they received the invitation and what intention it had.

*‘I could not read it, but I know English. I just guessed.’* (Woman, 38 years)

*‘No, I did not understand the purpose of the health assessment. I just received the invitation and my interpretation was that I had to go there.’* (Man, 35 years)

The information given in the letter of invitation was confined to date and place. The most obvious information that was missing was said to be the purpose of the health assessment. Several informants criticized the use of Swedish when communicating with newly arrived immigrants because they arrived at the health assessment not knowing what to expect.

The health assessment was considered by the informants to be a suitable opportunity to receive information about the Swedish healthcare system. However, no informant received such information and this likely contributed to the informants’ lack of trust and understanding of the healthcare system.

There seemed to be several missed opportunities for communication and the asylum seekers described how they themselves also contributed to these missed opportunities. They described how they did not have enough courage to interact during the health assessment, how they acted in a passive manner and how they just accepted and adjusted to the situation.

*‘I had so many doubts…I was thinking…maybe it* [the health assessment] *is regulated by law…I had to follow the law. If so, I have done it.’* (Woman, 38 years)

In spite of what they said was lacking, none of the informants would dissuade any fellow immigrant from attending the health assessment.

*‘Everything is relative, if I compare it with Eritrea. Here* [in Sweden] *at least we get healthcare that we didn’t get there* [in Eritrea]. *Thus one is satisfied even if it isn’t perfect.’* (Man, 35 years)

### Previous pictures

The informants said they felt compelled to leave their native country, since they felt there was no future. Old contexts and frames of reference were carried along to Sweden. Many of the informants in this study said that the thoughts and actions that would be rational in their old context were not applicable in their new context in Sweden and this added to the feeling of being in a state of limbo.

The informants had all grown up in and around the city of Asmara in Eritrea and had almost no experience of rural life. Only those who had been in the military service had experienced life in other parts of the country. Whether having been in the army or living a civilian life in Asmara, they all had experienced life in the shadow of the political and armed conflict that had been ongoing in the nation for several decades.

Asmara had clinics and hospitals that were sufficient for the people living in the capital, although access was said to vary, especially depending on one’s financial situation. There were also pharmacies, even though there was often a shortage of drugs. Preventive measures in relation to health were rare and were said by some to be confined to tests for HIV and vaccination programmes targeting children. Most of the informants had experiences with being targeted for information about HIV and vaccinations from public service messages on the radio and TV. Some had also received such information in the military service in addition to being tested for HIV.

*‘All who do military service are tested for HIV. They just informed us that they would carry out an HIV test. Those who tested positive were informed of this and then expelled from the military service in order not to infect others.’* (Man, 36 years)

Some of the informants said that testing for HIV was a comprehensive measure in Eritrea. This included pre-test information on the disease that described how it is a chronic but treatable disease and described the routes of transmission. They expressed how they felt well informed ahead of their HIV test in Eritrea, but they expressed disappointment over the lack of information in connection to their HIV test during their health assessment upon arrival in Sweden.

*‘One cannot compare Eritrea with Sweden, but there…in Eritrea…when testing for HIV…they give information about the disease. If one tests negative, they inform you that you don’t have the disease and tell you how to protect yourself in the future. I think that is good, but it wasn’t like that here in Sweden.’* (Woman, 36 years)

*‘In Eritrea, all of the people are gathered together for one hour before the blood test in order to get information about the disease. This is information on how the disease is spread, what happens to anyone testing positive, and how to protect yourself. They say it is an ordinary disease… almost.’* (Man, 36 years)

For some of the informants the migration process had started when they left what was said to be a never-ending military service. Others described their life in Eritrea as ‘normal’ and with no significant health-related burdens, but with no hope for a future in Eritrea. For some it took weeks, for others years, from crossing the Eritrean border to finally reaching a country where they could apply for asylum. The experience of being an undocumented immigrant in foreign countries, left to smugglers and risky travels by land and sea was said to be a threat that made them feel even more vulnerable.

*‘I came via Sudan. It was not easy. Smugglers helped us to cross the border. In fact…I do not want to talk about that time…I have been through a lot.’* (Woman, 43 years)

Even though they had experienced difficulties in their homeland that were significant enough to make them leave, the informants expressed that the time after their arrival in Sweden was very difficult. Their expectations for a better life were not met, at least not immediately. Instead they felt vulnerable, having turned their back on the past and trying to adjust to a society where they did not recognize themselves and where the values they previously took for granted could be questioned.

### Being subjected to the Swedish healthcare system, while in the asylum process

The time spent in the asylum-seeking process awaiting the final residence permit could last from a few weeks to several years. The informants described themselves and their existence as asylum seekers as being ‘in a state of limbo’, i.e. being somewhere ‘in between’ and awaiting asylum and security. They described how they had to move from one place to another staying in accommodation centres provided by the migration authority and, when possible, in the homes of relatives and friends. This period was accompanied with thoughts, worries and uncertainty about the outcome of their application for asylum and these were difficult to cope with.

*‘The time from submitting the application for asylum and awaiting the decision was the worst period. I had risked a lot to come here and I did not feel well. The only thing I had on my mind was the residence permit.’* (Man, 27 years)

The health assessment took place at a time when the asylum seekers were predominantly occupied with thoughts and worries about the outcome of their asylum application. They expressed how their main focus was on the legal process related to the asylum application and their thoughts about their own health and health-related needs became secondary.

In contrast to the fact that the health assessment is optional, several of the informants expressed how they thought it was mandatory.

*‘I received a letter in Swedish with the time for the assessment, and that made me interpret it as compulsory.’* (Man, 35 years)

Being subjected to the Swedish healthcare system was thus described as a multifaceted issue. The informants repeatedly reported that the health assessment was the first encounter between them and the Swedish healthcare system. They expressed uncertainty as to whether the health assessment would contribute to their health and their expectations for the health assessment seemed to be low.

The informants perceived that the focus of the health assessment was restricted to blood tests, and they expressed disappointment for not being given the opportunity to bring up their own health needs and concerns. This one-way communication contributed to uncertainty about whether they would get help and treatment if they were diagnosed HIV positive and to what extent an identified disease would impact their ability to stay in Sweden.

*‘Of course, I have been thinking … if they find something, how will that affect my chance to obtain asylum, and if they find a disease, will they help or what will happen?’* (Woman 36 years)

*‘I have an allergy and I asked for help to get in touch with a dermatologist. After some days I received a letter saying that I was not entitled to such help as an asylum seeker.’* (Woman 28 years)

The use of interpreters, either on the phone or present in the clinic, seemed to be a common practice. However, there were times when no interpreter had been engaged at all or only an interpreter using another language than that of the immigrant was engaged.

*‘There was no interpreter. I only know a little English. In fact, it was not easy. It was very difficult for me. Next time at the clinic I was offered an interpreter in Arabic. ‘Maybe you know Arabic,’ they said. ‘A little,’ I said. It was better than nothing.’* (Woman, 30 years)

Interpreters are intended to reduce difficulties in relation to communication, but the informants described how this did not eliminate the feeling of fear. On the contrary, communicating via an interpreter was not always regarded as safe because they did not always trust the interpreter’s confidentiality.

*‘In spite of the fact that there was an interpreter, I did not dare to ask about all I wanted to.’* (Woman, 28 years)

However, the same feeling of fear was expressed by another informant when there was no interpreter present at all.

*‘I needed an interpreter, but there was no interpreter. Indeed, it was not easy and I was scared.’* (Woman, 30 years)

### Ambiguity and mistrust

To be in a state of limbo was described as being in-between, vulnerable, and powerless. The informants had left difficult times in their country of origin and arrived in their new country in a state of mistrust. The asylum seekers expressed a sense of ambiguity and mistrust both in their meetings with the migration authority and with the Swedish healthcare system. Uncertainty and a sense of insecurity were expressed regarding what role the migration authority had in relation to the healthcare system.

*‘It is the migration authority…on their assignment the health assessment was carried out… and the migration authority wants to know* [the results]. *It was so stressful.’* (Woman, 38 years)

Ambiguity on what to say or do to improve the likelihood of receiving asylum led the asylum seekers to act in a passive manner and to keep a low profile. Under such conditions their ability to act as active human beings diminished. This in turn led them to feel objectified.

Perceiving the health assessment as mandatory combined with not knowing the intention of the health assessment and the inability to communicate in a common language was said to create feelings of difficulty, insecurity and fear. It was a common belief among the informants that the outcome of the health assessment was communicated to the migration authority and that these results could influence their chances of obtaining a residence permit. The possibility of such a link was a significant source of worry and anxiety.

Questions similar to those being asked by the migration authority officer were addressed during the health assessment. This was interpreted by the informants to be a double check of the information that had been declared earlier to the migration authorities.

*‘I went to the health center and it was like an interrogation, “Do you speak English? Do you speak any other languages? When did you come to Sweden? Why did you come here?” It was all so stressful! It was the same as they asked me at the migration authority.’* (Woman, 38 years)

Feelings of difficulty, insecurity and fear were also said to be linked to an impersonal attitude from the healthcare personnel. Limited dialogue and restricted communication were problems regardless of whether an interpreter was present or not.

*‘I don’t have much to say, but I wanted them to give me more information. And to listen to me.’* (Woman, 43 years)

*‘This feeling of fear was present all the time. Without explaining anything, she did the blood test.’* (Woman, 28 years)

The informants did not know what tests had been carried out during the health assessment. Although HIV tests were carried out routinely, none of the informants was able to confirm that informed consent had been given.

*‘No, they did not say anything about HIV.’* (Woman, 38 years)

*‘They did not ask. They just took the blood, three times, without saying anything.’* (Man, 27 years)

Several informants said that they thought that an HIV test had been done, but they did not know for sure. In contrast, it was more common to know that a test for TB had been carried out because they were told to come back to the clinic after 3 days to get the result.

*‘I did not receive much information. She explained about the TB test, but I got no information in relation to the blood test. I still do not know.’* (Woman, 28 years)

A common story told by the informants was that they experienced the health assessment as focusing on TB screening and HIV testing and that this was carried out in a routine manner with no or limited information and communication ahead of time. When one informant came back for the 3-day follow-up of the TB test, only then did she learn that she also had had an HIV test done.

*‘Regarding the TB, she said, there is nothing. It is negative. “And then we also checked HIV”, she added. It was also negative. Of course I was shocked. She said nothing, until afterwards…’* (Woman, 36 years)

*‘Of course, it is inappropriate not to tell why a blood test is done. Without information and communication, I was not able to be a part of my own health assessment.’* (Woman, 28 years)

Some informants claimed that it is important to know what actual tests have been carried out and that it is equally important to know the results. Some of the informants were told that only positive test results would cause the clinic to contact the individual again, but it was said that sometimes negative results had also been conveyed. This inconsistency, in combination with poor communication, e.g. writing in Swedish, made the individual unaware of the test results and created confusion, distrust, and fear.

*‘The result came in a letter, in Swedish. I tried to read it but understood nothing. I still do not know’* (Man, 27 years)

### Adjustment

Being in the asylum-seeking process and bound by legal constraints means that there are few options and only limited choices to make. As several informants shared, they perceived themselves to be subjected to circumstances and situations that they could not influence. The informants expressed sentiments of hopelessness and a lack of real power to act or change the situation they were in.

*‘What could I do but to comply, I had no choice.’* (Man, 27 years)

The informants further expressed that the asylum-seeking process seemed to be a procedure that could not be altered or influenced by the individual. No informant articulated any notion on rights, only on how to comply with the system with the ultimate purpose in mind, to obtain a residence permit. Because the health assessment was seen as part of the asylum-seeking process and linked to the legal aspects of the process, the informants described how it was logical to maintain an attitude of obedience and compliance in this area.

Instead of demanding information in their own language, information on the purpose of the health assessment and the right to articulate their own health-related needs, they resigned themselves and turned to compatriots who had arrived earlier in Sweden for help with interpreting information. The consequences of such consultations could lead to problems for some immigrants because they received misinformation. This was exemplified by one informant who left the clinic after having been tested for HIV, knowing that if the clinic did not contact him again, then the test result was favourable. He was told by a fellow immigrant, however, that he had AIDS because his blood test result was ‘negative’. Of course, in a medical context a negative test result means that the patient does not have the disease in question.

*‘I received a letter from the clinic, in Swedish. I went for help from a person who arrived in Sweden before me, a language student, thinking that he might be able to help. He made a wrong translation, saying that I had AIDS. I was terrified. I gave the letter to another person who said it was negative. I was so worried. I should not have gone to the first one, but to whom should one turn?’* (Man, 36 years)

## Interpretation of data and discussion

### Few asylum seekers attend the health assessment

The rationale for this study was based on the limited knowledge of why relatively few asylum seekers in Sweden attend the offered health assessment. In an attempt to fully understand the reasons for the low attendance, the focus was mainly to understand how the former asylum seekers themselves perceived the health assessment. Understanding the reasons for this is crucial when designing future routines for providing healthcare for asylum seekers.

The informants of this study all attended the health assessment. They had all received an invitation and they had all perceived the health assessment to be mandatory. This explains, at least to some extent, their attendance. It could also be that the informants attended the health assessment as a way to know their own health status.

When the asylum seekers do not come for the health assessment, healthcare professionals sometimes assume in a rather simplistic way that reasons for not attending are that they do not want to come. Thus, not attending might be seen as the asylum seekers not taking full responsibility for their own health, when in fact the reasons for not attending could well be structural and caused by the Swedish healthcare system itself.

### The invitation

The content of the invitation is regulated by national guidelines that state that the invitation should include information on the voluntary aspects of the health assessment [6]. Ensuring asylum seekers autonomy in relation to the healthcare services reflects the importance this is given by the Swedish Government. The health assessment is, on the other hand, an important systematic attempt by which the government can control infectious diseases among asylum seekers in Sweden. Explicitly stressing the voluntariness contradicts this goal. Thus, this seems like a paradox, that the Swedish health authorities want to control infectious diseases, but do not want anyone to feel like they have to participate in it. This contradiction becomes even more problematic when considering that, in no other part of the healthcare system, explicit information on voluntariness is expressed since the general principle in Sweden is that all healthcare, with few exceptions, is optional. Further, it might be assumed that the optional nature of the offer hides a message that the invitation is not a promise to be given treatment should a disease be detected, since medical treatment for asylum seekers is confined to immediate attention that cannot be deferred. In the eagerness to ensure asylum seekers’ autonomy in relation to the healthcare services, it seems that they are looked upon as if they cannot make their own decisions. It could be argued that asylum seekers do not need to be treated differently in this matter. They need to have enough information about the issue concerned, in order for them to make an informed decision in the best interest of themselves. The data from this study reveal that, whether optional or voluntary, the health assessment is looked upon as a positive offer.

### Poor communication and asymmetry

Many asylum seekers in Sweden come from cultures where authorities are regarded as representing power and where communication from any authority will be perceived as a demand and therefore not be questioned [22]. Similarly, when receiving the invitation from the healthcare system, attendance was perceived as mandatory. Even if Swedish culture and value systems differ significantly compared to many other countries [22], hierarchical structures are still found in the Swedish healthcare system. This in turn leads to an imbalance or asymmetry in the encounter between the doctor or nurse and the patient and such a relationship often contradicts mutual communication. The concept of asymmetry has been discussed by Parsons [23], who implies that healthcare personnel, for example, are seen as experts who possess professional attributes and advanced technical competence in contrast to patients, who are seen as passive and who become dependent on the experts. The same asymmetry could be seen with regard to the situation of health assessments and this might have contributed to the poor communication during the health assessment.

Hierarchical structures, by definition, do not encourage questioning or encounters on equal terms. In order to cope with this situation, the informants said that they just had to accept and adjust. The data in this study showed that insufficient communication between healthcare professionals and asylum seekers influenced the health assessment in a negative way. Language is one crucial barrier between healthcare personnel and asylum seekers [24,25]. The use of professional interpreters might overcome this barrier but, as this study showed, they were not always engaged or they might not be qualified for the language needed.

The informants expressed how they felt being treated as objects by the healthcare professionals and how they had difficulties in understanding the purpose of the health assessment. It seems as if the asylum seekers’ own perceived health needs were not met during the health assessment. They might have felt other concerns but the focus was felt to be restricted to contagious diseases.

To reduce the asymmetry to some extent in communication [23], the main responsibility should lie on the more powerful part, the healthcare system itself. Improved mutual communication would probably enhance the quality of the encounter between the care giver and the asylum seeker.

### A limbo – between past and present

The life lived in the past will always act as a frame of reference and influence the view of the present. By contrasting the past with the present, the informants attributed things to be better or worse in Eritrea or in Sweden. In a Swedish study, the asylum seekers were shown to carry with them cultures and traditions from their respective homelands [26]. Facing a new and unfamiliar context could be difficult because Swedish culture represents other values, such as secular-rational values in contrast to traditional religious values and favouring self-expression values in contrast to survival values [22]. Thus, besides language, cultural differences might be other crucial barriers to overcome in order to create a ‘true’ encounter [25]. Difficulties in bridging the gap between different cultures might result in strong feelings. Upon arrival in Sweden and in the meeting between their previous and their new contexts, participants expressed feelings of mistrust, ambiguity and fear and indicated a feeling of being in a state of limbo.

The concept of liminality – which is known colloquially as limbo – was used by the social anthropologist Arnold van Gennep to describe the middle of the three phases of rites of passage: separation; margin (or liminal); re-aggregation [27]. This was later developed by another anthropologist, Victor Turner, who developed the notions of liminality and of being ‘in between’ in his work on rituals. He observed that liminal people are suspended in social space, i.e. a social limbo [28]. Liminal people are at a threshold outside the boundaries of society; they have ‘been declassified but are not yet reclassified: they have died in their old status and are not yet reborn in a new one [29].

### A need for improvements

The informants in this study claimed that they were initially given a lot of formal information at the same time and that information about the health assessment was only one small part of this. This might explain why they felt they had not received sufficient information ahead of the health assessment in order to understand the purpose of the health assessment. Obviously, this had a negative impact on their first encounter with the healthcare services, but this might also influence future contacts in a negative way. Healthcare services should be carried out respectfully and if targeting asylum seekers they should also be carried out in a culturally sensitive way. It is thus not enough that healthcare services simply are provided. They should also be accessible, acceptable and be of a good quality [30].

The health assessment offered, might be interpreted as a way of materializing the fundamental right to health [31]. As such an important issue, all efforts have to be made to reduce the missed opportunities to communicate that were identified in this study.

#### Methodological aspects

Because this is a qualitative research study where the informants have been selected purposively, statistical generalizations cannot be made from the findings. The constructed model could, however, be adjusted and used in similar situations for shaping policies, instructions and practice within the field of immigrant health, particularly when it comes to issues related to health assessments or screening programmes targeting immigrants. The limitation due to language and translation problems was partly balanced by the use of a specially trained interpreter whose mother tongue is Tigrinya. Nevertheless, information might have been lost in the translation process.

## Conclusions and implications

The reason for asylum seekers’ low attendance for the health assessment is most likely not that they do not accept the offer. It is instead likely that they do not attend the health assessment due to structural weaknesses related to poor communication. Improved communication targeting asylum seekers by migration authorities and healthcare providers is, therefore, vitally important. Providing healthcare services in an accessible and acceptable way to asylum seekers will probably lead to greater participation in the health assessments.
